# Analysis of pharyngeal microbiome characteristics in HIV-infected individuals: correlation between the degree of immunosuppression and microbial dysbiosis

**DOI:** 10.1186/s12879-026-13075-2

**Published:** 2026-03-24

**Authors:** Xiaolei Ji, Yuanyuan Geng, Chen Guo, Shu Zhang, Hongge Li, Chen Li, Yu Du, Xiaonan Guo, Beibei Miao, Yijie Hu, Jialin Lv, Zixing Zhou, Jie Gong, Yihua Sun

**Affiliations:** 1 Key Laboratory of Medicine, Nantong Center for Disease Control and Prevention, No.189 Gongnongnan Road, Chongchuan District, Nantong, Jiangsu 226001 China; 2 National Key Laboratory of Intelligent Tracking and Forecasting for Infectious Diseases, National Institute for Communicable Disease Control and Prevention, Chinese Center for Disease Control and Prevention, 155 Changbai Road, Changping District, Beijing, 102206 China; 3 Nantong Chongchuan Center for Disease Control and Prevention, No.47 Zhongxiuzhong Road, Nantong, Jiangsu 226001 China; 4 Xuzhou Center for Disease Control and Prevention, 142 West Erhuan Road, Quanshan District, Xuzhou, Jiangsu 221000 China; 5 Suzhou Center for Disease Control and Prevention, 16 Guangqian Road, Xiangcheng District, Suzhou, Jiangsu 215137 China; 6 College of Life Sciences, Zhejiang Normal University, 688 Yingbin Avenue, Jinhua, Zhejiang 321004 China; 7 College of Public Health, North China University of Science and Technology, No. 21 Bohai Avenue, Caofeidian New City, Tangshan, Hebei 063210 China; 8 School of Public Health, Chengdu University of Traditional Chinese Medicine, Chengdu, Sichuan 611137 China

**Keywords:** HIV, Pharyngeal microbiome, Immunosuppression, 16S rRNA sequencing

## Abstract

**Objectives:**

Despite the significant improvement in survival rates due to antiretroviral therapy, opportunistic infections continue to pose a major health risk for individuals living with HIV. As a key anatomical intersection of the upper respiratory and digestive tracts, the pharyngeal microbiome during HIV infection remains poorly characterized. This study aimed to compare the composition of bacterial and fungal communities in the pharynx between HIV-infected individuals and healthy controls, and to investigate their association with host immune status.

**Methods:**

Throat swab samples were collected from 70 HIV-infected individuals—stratified into severe, moderate, and no/mild immunosuppression groups based on CD4⁺ T-cell counts—and 18 healthy controls. Microbial composition was assessed using 16S rRNA and internal transcribed spacer (ITS) amplicon sequencing.

**Results:**

Bacterial alpha diversity was significantly reduced in the severe immunosuppression group (CD4⁺ T cells < 200/µL) compared to other groups, whereas fungal alpha diversity did not differ significantly across groups. Beta-diversity analysis revealed a distinct bacterial community structure in the severe immunosuppression group. Fungal communities, however, differed significantly between all HIV-infected individuals and healthy controls. At the genus level, the severe immunosuppression group exhibited a decrease in commensal bacteria such as *Prevotella* and an increase in *Halomonas*. Additionally, *Candida* was significantly enriched in the severe immunosuppression group, while *Exophiala* levels were elevated across all HIV-infected groups.

**Conclusions:**

The pharyngeal microbiome of HIV infected individuals undergoes significant changes, with bacterial community disorder associated with immune suppression, while the fungal community changes are more closely related to the HIV infection status of the individuals. The enrichment of Candida in individuals with severe immune suppression is more appropriately interpreted as a manifestation of fungal dysbiosis associated with advanced immunosuppression and a potential indicator of increased susceptibility to oropharyngeal candidiasis, rather than as a diagnostic biomarker of overt disease.

**Clinical register number:**

Not applicable.

**Supplementary Information:**

The online version contains supplementary material available at 10.1186/s12879-026-13075-2.

## Introduction

The widespread adoption of antiretroviral therapy (ART) has substantially reduced AIDS-related mortality and prolonged survival in people living with HIV [1]. Nonetheless, immune recovery is frequently incomplete. Sustained immunodeficiency or functional impairment—especially among individuals diagnosed at advanced stages or with suboptimal immune reconstitution—maintains a high risk of opportunistic infections [2, 3]. These infections, caused by pathogens such as *Pneumocystis*, *Mycobacterium tuberculosis*, *Candida*, and *Cryptococcus*, continue to represent a major cause of hospitalization, clinical deterioration, and mortality in HIV-infected populations, significantly impairing long-term quality of life and clinical outcomes.

Emerging evidence indicates that HIV infection is associated with dysbiosis across multiple mucosal sites, including the gut, lungs, and oral cavity [4, 5]. For example, oral colonization by *Candida* is commonly increased in HIV-positive individuals [6], whereas immunosuppression in the lungs promotes the expansion of opportunistic fungi such as *Pneumocystis* and *Talaromyces marneffei* [7, 8]. Such microbial alterations may further compromise mucosal immunity and epithelial barrier function, thereby accelerating disease progression and elevating susceptibility to opportunistic infections [9].

Current knowledge of the respiratory microbiome in HIV infection remains limited in several key aspects. First, most studies have focused on individual microbial kingdoms rather than providing an integrated profile of the whole pharyngeal microbiota following HIV infection. Second, although the lower respiratory tract and gut have been relatively well characterized [10, 11], the upper respiratory tract—specifically the pharynx—has been underexplored [12].

Located at the intersection of the respiratory and digestive tracts, the pharynx is continuously exposed to environmental pathogens and airborne particulates and serves as a critical hub for mucosal immune activity. Its resident microbiota contributes importantly to the maintenance of epithelial barrier integrity, regulation of local immune homeostasis, and defense against pathogen invasion [13, 14]. Under conditions of severe immunosuppression, the pharyngeal microbiota is particularly susceptible to dysbiosis, potentially becoming a reservoir for opportunistic pathogens. This is especially evident in patients with profound depletion of CD4⁺ T cells, where microbial community stability is more likely to be disrupted [15]. Therefore, elucidating the overall composition of the pharyngeal microbiome in HIV-infected individuals and delineating its divergence from that of healthy controls are essential for understanding how HIV-associated immune deficiency reshapes the respiratory microbiota and increases the risk of opportunistic infections [13].

To address these gaps, we performed a cross-sectional study utilizing internal transcribed spacer (ITS) and 16S rRNA gene amplicon sequencing to systematically analyze throat swab samples from 70 HIV-infected individuals and 16 healthy controls. This study aimed to comprehensively characterize differences in bacterial and fungal communities between these groups, evaluate whether HIV infection drives significant alterations in the pharyngeal microbiota, and establish a foundation for subsequent mechanistic inquiry and potential clinical interventions.

## Methods

### Patients

In May 2025, we conducted the preliminary assessment of 82 HIV infected individuals at Nantong Third People’s Hospital (Nantong Infectious Disease Hospital). The inclusion and exclusion criteria are shown in Table 1. After screening, a total of 12 infected individuals who did not meet the criteria were excluded, and ultimately 70 eligible patients were included as the infection group. In addition, this study recruited 18 HIV negative healthy volunteers from the local disease and prevention control center as the control group. All participants completed a standardized questionnaire on smoking status, alcohol use, oral hygiene practices (including tooth-brushing frequency and use of mouthwash), recent (within the previous 3 months) systemic antibiotic or antifungal use before sample collection, and dietary habits, and underwent a brief oral examination to assess periodontal status; these behavioral and oral health characteristics, including recent antimicrobial exposure, were comparable between the HIV infected groups and healthy controls (Table 2). This research plan has been approved by the Ethics Committee of Nantong Center for Disease Control and Prevention (No.: Nantong CDC Lunzi [2025] No. 23). All participants signed a written informed consent form in accordance with the Helsinki Declaration.

**Table 1:** Inclusion and exclusion criteria

Category	Criteria
​Inclusion Criteria​	a) Age ≥ 18 years, without psychiatric disorders or cognitive impairment, and capable of completing questionnaires independently;
	b) Positive HIV antibody confirmation test;
	c) Already initiated antiretroviral therapy (ART).
​Exclusion Criteria​	a) Presence of acute respiratory symptoms;
	b) No record of CD4⁺ T-cell count or viral load testing within the past 3 months;
	c) Unwillingness to participate in the study.

### Sample collection and preservation

Pharyngeal swab samples were collected using commercially available sampling tubes containing a preservation solution suitable for both bacterial and fungal nucleic acid stabilization. Collection was performed by trained clinical personnel under standardized operating procedures. Immediately after collection, samples were stored at 4 °C and transported to the laboratory within 2 h. Prior to DNA extraction, all samples were transferred to and maintained at − 80 °C in an ultra-low temperature freezer.

### DNA extraction

Each sample was vortexed for 1 min to homogenize the contents, after which 1 mL of the suspension was transferred to a 1.5 mL microcentrifuge tube. The tube was centrifuged at 12,000 rpm and 4 °C for 10 min, and the supernatant was discarded. Following cell wall disruption using an established protocol [16], total DNA was extracted with the QIAamp DNA Mini Kit (Qiagen, Hilden, Germany). The extracted DNA was stored at − 80 °C until further processing.

### Library preparation and sequencing

Using the extracted DNA as template, the V3–V4 hypervariable regions of the 16S rRNA gene and the ITS region were amplified by PCR with the primer pairs 338 F/806R and ITS1F/ITS4R, respectively. Amplification products were purified via 2% agarose gel electrophoresis using a PCR Clean-Up Kit (Zhonggao Yuhua Biotechnology Co., Ltd., Shanghai, China) and quantified with a Qubit 4.0 Fluorometer. Sequencing libraries were prepared with the NEXTFLEX Rapid DNA-Seq Kit (Blue scape, Beijing, China) following the manufacturer’s instructions, which included adapter ligation, size selection with magnetic beads, PCR enrichment, and final library purification. Libraries were subsequently sequenced on the Illumina Nextseq2000 platform.

### High throughput sequencing

The raw 16S rRNA and ITS amplicon sequencing data were processed separately but using analogous pipelines. After demultiplexing, fastp [17] was used for quality control, removing low-quality reads, reads containing ambiguous (“N”) bases, and sequences shorter than 50 bp. Paired-end reads were merged with FLASH, with a minimum overlap length of 10 bp and a maximum overlap mismatch rate of 0.2. Samples were demultiplexed according to barcodes and primers (no barcode mismatches allowed and up to 2 primer mismatches). Within QIIME2 [18], the DADA2 plugin was used to denoise both 16S and ITS reads and infer amplicon sequence variants (ASVs) [19]. For each dataset, per-base quality score profiles were inspected and dataset-specific trimming and truncation parameters were chosen: low-quality bases at the 5′ and 3′ ends were removed, reads were truncated at positions where quality scores consistently dropped, and reads with excessive expected errors were discarded. Singleton ASVs (total count of 1 across all samples) were removed. Putative chimeric sequences were identified and removed using the removeBimeraDenovo function in DADA2 with the “consensus” method, applied separately to the 16S and ITS datasets. Because ITS amplicons exhibit greater length variability than 16S rRNA fragments, the ITS workflow used less aggressive 3′-end truncation to retain variable-length ITS sequences while still removing low-quality tails; aside from these ITS-specific trimming settings, all subsequent steps in the pipeline (denoising, chimera removal, ASV inference, and downstream analyses) were identical for the 16S and ITS data. Rarefaction analyses based on the ASV table were performed to assess whether sequencing depth per sample was sufficient to capture community diversity, and the resulting rarefaction curves are presented in Supplementary Figure S1. Importantly, statistical comparisons of α-diversity, β-diversity, and differential taxon abundance were conducted on non-rarefied data (relative abundance tables), and no hypothesis testing was performed on rarefied datasets.

Silva database (v138) and UNITE database (v9) were used for taxonomic annotation of ASVs. Relative abundances were then calculated at the ASV, genus, and phylum levels; unless otherwise specified, community composition and differential abundance results are presented at the genus level, and all detected bacterial and fungal taxa were included in the statistical analyses, with bar plots displaying the top 10 genera in each group and the remaining taxa collapsed into “Others”.

### Statistics

Mothur (http://www.mothur.org/wiki/Calculators) was used to calculate the α-diversity indices, including Chao1 (richness) and Shannon (diversity). The normality of continuous variables was assessed using the Shapiro-Wilk test. Normally distributed variables are presented as mean ± standard deviation (SD), and comparisons between groups were analyzed using one-way analysis of variance (ANOVA). Non-normally distributed variables are presented as median and interquartile range [M (IQR)], and group comparisons were performed using the Kruskal-Wallis H test. Categorical variables are presented as number and percentage [n (%)] and were compared using the Chi-square test. For comparisons across three or more groups, if the Kruskal-Wallis H test or ANOVA indicated a significant difference, a post-hoc Tukey-Kramer test was applied for pairwise comparisons to control for type I error. β-diversity analysis was performed using principal coordinate analysis (PCoA) based on Bray-Curtis distance, and permutational multivariate analysis of variance (PERMANOVA) was used to test the significance of differences in community structure between groups. All statistical analyses were performed using R language (v4.3.1). To account for potential confounding by behavioral and environmental factors, multivariable PERMANOVA models for β-diversity and multivariable differential abundance analyses were performed with smoking status (current vs. non-current), oral hygiene practices (tooth-brushing ≥ 2 times/day vs. <2 times/day), recent (within the previous 3 months) systemic antibiotic or antifungal use (yes/no), dietary pattern (higher fiber vs. higher fat intake, as assessed by questionnaire), and periodontal status (no/mild vs. moderate/severe periodontitis) included as covariates. For all analyses, a two-sided *p* < 0.05 was considered statistically significant. Differential abundance testing was performed on the relative abundances of all detected taxa (at the genus level for both 16S and ITS; and at the species level when reliable assignments were available) across study groups. To control false positives due to multiple comparisons, p-values from these taxon-wise tests were adjusted using the Benjamini–Hochberg false discovery rate (FDR) procedure within each microbial domain (bacteria vs. fungi) and taxonomic level, and only taxa with FDR-adjusted *p* < 0.05 were interpreted as significantly different.

## Results

### Characteristics of the study objectives

The current study included 70 HIV infected individuals and 18 HIV negative healthy controls (HC). According to the immune staging criteria of the World Health Organization (WHO), HIV infected individuals were divided into three groups based on CD4 ⁺ T cell count: severe immunosuppression group (CD4 ⁺ T cells < 200/µ L, *n* = 21), moderate immunosuppression group (CD4 ⁺ T cells 200–499/µ L, *n* = 26), and no/mild immunosuppression group (CD4 ⁺ T cells ≥ 500/µ L, *n* = 23). The mean ages of each group were 49.7 ± 11.4 years, 46.8 ± 14.0 years, 48.9 ± 13.5 years, and 43.3 ± 6.5 years, respectively (*p* = 0.307). The proportion of males in the gender composition was 81.0%, 88.5%, 78.3%, and 77.8%, respectively (*p* = 0.759), and there was no statistical difference for the baseline information among the groups. Similarly, behavioral and environmental factors known to influence the oral and pharyngeal microbiome, including current smoking status, oral hygiene practices, recent systemic antibiotic or antifungal use, dietary habits, and periodontal status, were broadly comparable across the four groups (all *p* > 0.05; Table 2).

In the HIV infected group, the median CD4 ⁺ T cell counts were 80.0 (IQR: 30.0-117.0), 370.5 (307.3–429.0), and 747.0 (584.5-846.5) cells/µ L, respectively. The median viral load in the severe immunosuppression group was 60.2 copies/mL (IQR: 0-135000), while the viral load of the moderate and no/mild immunosuppression groups were all below the detection limitation.

All HIV infected individuals are currently receiving antiretroviral therapy (ART) (Table 2). However, the size of the healthy control group (*n* = 18) was smaller than that of the HIV-infected cohort (*n* = 70), which may have reduced statistical power, particularly for β-diversity and differential abundance analyses, and this imbalance should be considered when interpreting our findings. In addition, detailed clinical information on ART, including duration of treatment, specific regimens, adherence, and history of virologic failure, was not collected in this study, which may confound the interpretation of microbiome differences between groups.

**Table 2:** Participant characteristics​

Index	Group1 (CD4 < 200)	Group2 (200 < CD4<500)	Group3 (CD4 > 500)	HC	*p*-value
**Age**, mean (SD), years	49.7 (11.4)	46.8 (14.0)	48.9 (13.5)	43.3 (6.5)	0.307
**Gender**					0.759
Male, n	17	23	18	14	
Female, n	4	3	5	4	
**CD4 + T cell count**, median (IQR), cells/µL	80.0 (30.0–117.0)	370.5 (307.3–429.0)	747.0 (584.5–846.5)	ND	ND
**Plasma viral load**, median (IQR), copies/mL	60.2 (0–135000)	0 (0–0)	TND	ND	ND
**Treatment with ART**, %	100%	100%	100%	NA	ND
**Current smoker**, n (%)	7 (33.3%)	8 (30.8%)	6 (26.1%)	5 (27.8%)	0.942
**Brush teeth ≥2x/day**, n (%)	15 (71.4%)	18 (69.2%)	18 (78.3%)	14 (77.8%)	0.877
**Recent (≤ 3 months) systemic antibiotic use before sampling**,** n (%)**	3 (14.3%)	2 (7.7%)	1 (4.3%)	1 (5.6%)	0.538
**Recent (≤ 3 months) systemic antifungal use before sampling**,** n (%)**	2 (9.5%)	1 (3.8%)	1 (4.3%)	0 (0%)	0.481
**Moderate/severe periodontitis**, n (%)	6 (28.6%)	7 (26.9%)	5 (21.7%)	3 (16.7%)	0.821

Note: SD: standard deviation; TND: Target not detected; ND: Not done; NA: Not applicable; IQR: interquartile range. Intergroup comparisons were conducted using chi-square test for categorical variables and Kruskal-Wallis H test for continuous variables. Behavioral and oral health characteristics were assessed via questionnaire and brief oral examination. “Recent antibiotic use” and “Recent antifungal use” refer to systemic treatment within the 3 months preceding pharyngeal swab collection

### Diversity analysis of the upper respiratory tract microbiota

**Fig. 1:**
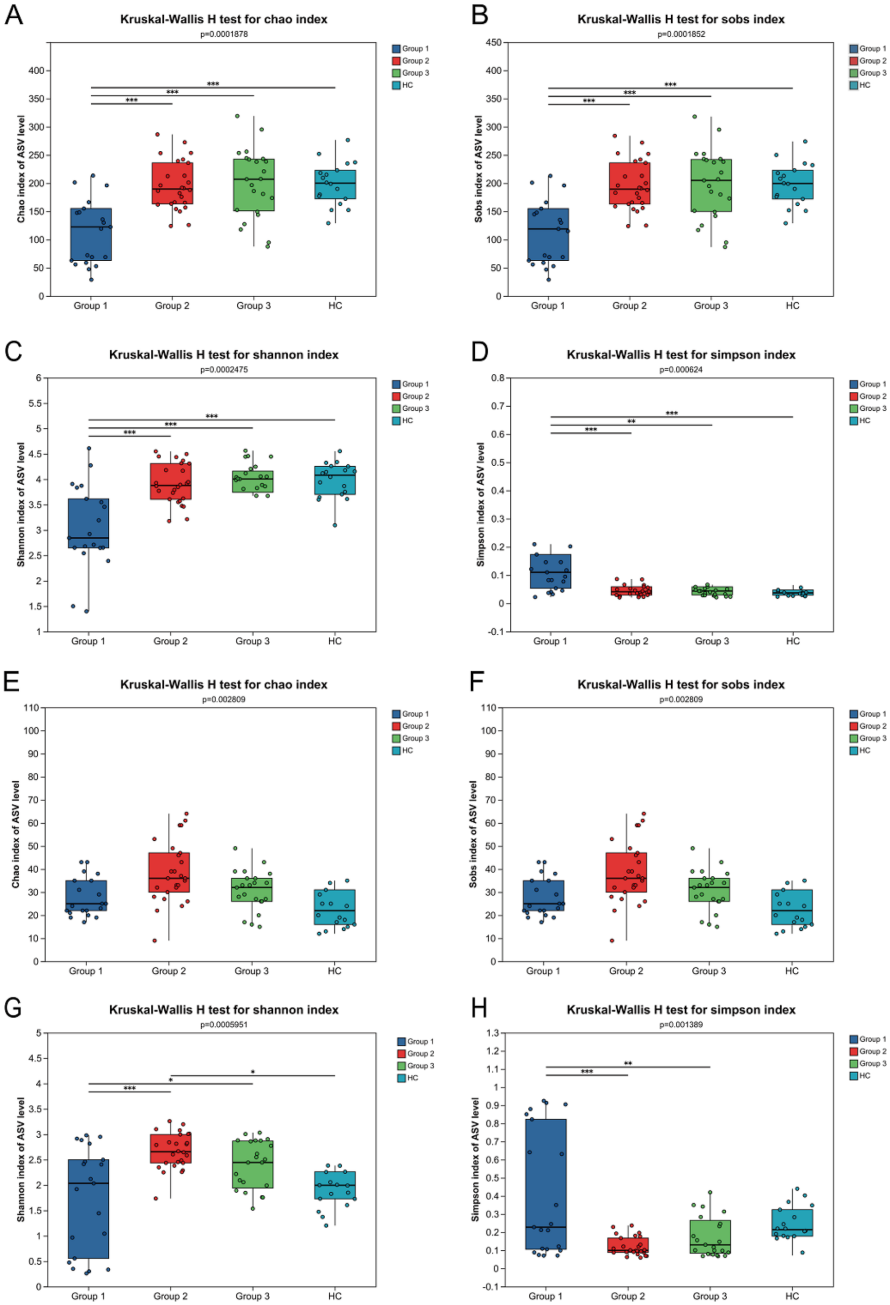
Comparison of α-diversity of upper respiratory microbiota between HIV infected individuals with different immune states and healthy controls. **A**-**B**: The richness indices of bacterial communities based on ASV levels (Sobs and Chao) were presented separately; **C**-**D**: The bacterial diversity indices based on ASV levels were presented separately (Shannon and Simpson); **E**-**F**: The richness indices of fungal communities based on ASV levels (Sobs and Chao) were presented separately; **G**-**H**: The fungal community diversity indices based on ASV levels were presented separately (Shannon and Simpson); The box plot in the figure shows the distribution of index values for each group. The significance of inter group differences was analyzed using Kruskal-Wallis test followed by Tukey-Kramer post-hoc test. The significance markers in the figure are derived from pairwise comparisons after the fact, and the significance levels are defined as: 0.01 < *P* ≤ 0.05 marked as: *P* < 0.05, *P* < 0.01, *P* < 0.001; *, 0.001 < *P* ≤ 0.01 marked as **, *P* ≤ 0.001 marked as ***

To further evaluate the differences in species abundance among different groups, we analyzed the α-diversity index of the four groups (Fig. 1). Among them, Sobs index and Chao index are used to reflect the richness of upper respiratory microbiota, while Shannon index and Simpson index are used as comprehensive diversity indices, taking into account both the richness and evenness of the community.

In terms of bacterium, the richness of bacterial community in Group 1 was significantly lower than that in Group 2, Group 3, and the HC group (*P* < 0.05; Fig. 1A and B). For the microbial diversity, the diversity of bacterium in Group 1 was significantly lower than Group 2, Group 3, and HC groups (*P* < 0.05; Fig. 1C and D). In terms of fungal, there was no significant difference in microbial richness among the four groups (Fig. 1E and F). The diversity of fungal in Group 1 was significantly lower than that of Group 2 and Group 3, but there was no statistical difference between the three infection groups and the HC group (Fig. 1G and H). Rarefaction curves for both bacterial and fungal ASV counts approached saturation for most samples, indicating that sequencing depth was sufficient to capture the majority of community diversity (Supplementary Figure S1).

**Fig. 2:**
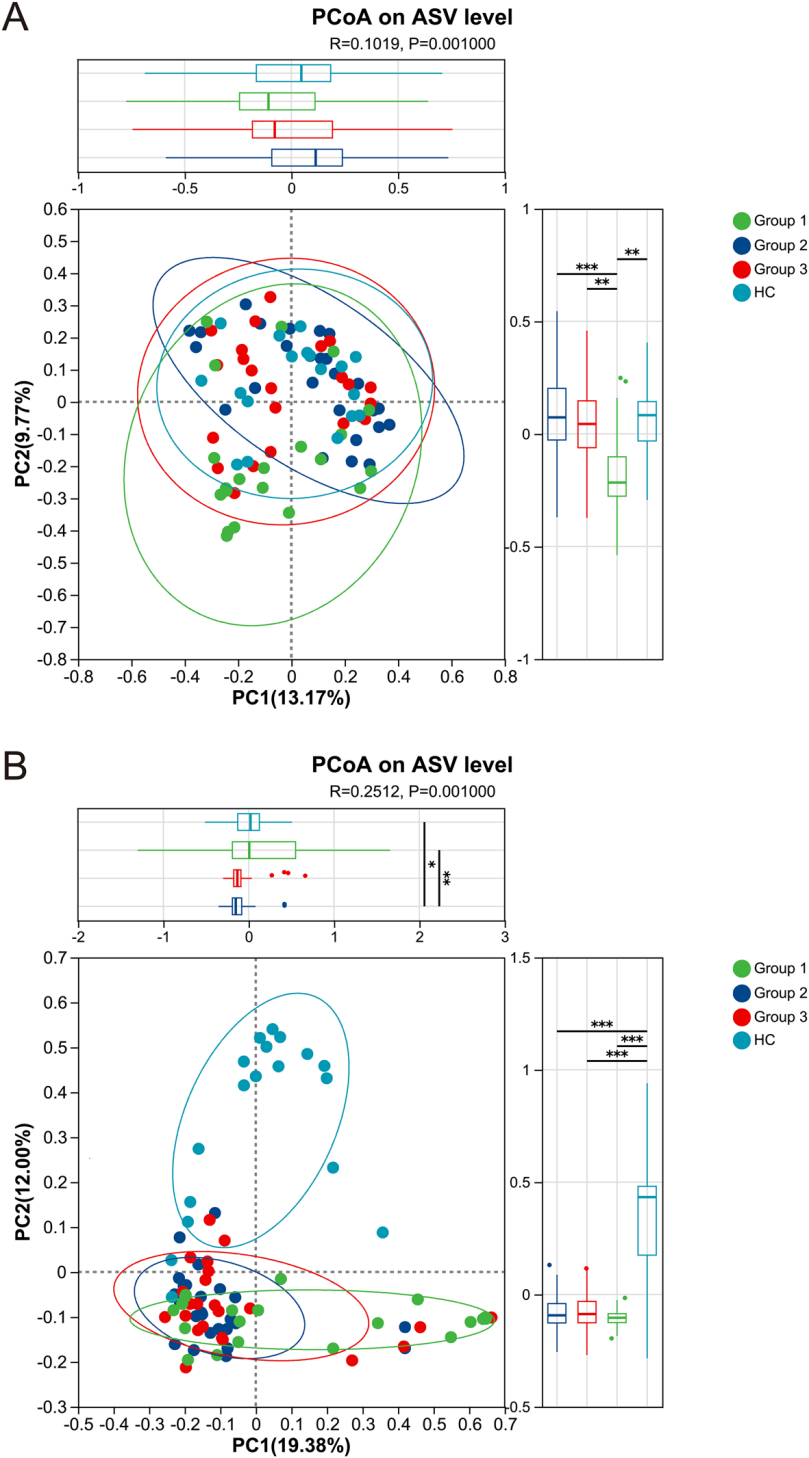
Results of PCoA analysis of upper respiratory microbiota β-diversity in HIV infected individuals with different immune states and healthy controls. **A**: PCoA of upper respiratory tract bacterial communities based on ASV level and Bray-Curtis distance algorithm. **B**: PCoA of upper respiratory fungal communities based on ASV level and Bray-Curtis distance algorithm. The percentage on the coordinate axis represents the explanatory power of the principal coordinate component on the total variation between samples. The differences between groups were tested using ANOSIM analysis, and the differences in bacterial community structure between groups were significant (*R* = 0.1019, *P* = 0.001), while the differences in fungal community structure between groups were significant (*R* = 0.2512, *P* = 0.001)

To understand the overall differences in the community structure of upper respiratory tract microbiota among different groups, we conducted principal co coordinates analysis (PCoA) based on bacterial and fungal ASV levels and Bray-Curtis distance algorithm. The analysis results of bacterial community structure are shown in Fig. 2A, where the two principal axes PC1 and PC2 show 13.17% and 9.77% of the overall variation, respectively. The scatter plot visually shows that the distribution of sample points between Group 1 and the other three groups shows a trend of separation. At the same time, the box plot of the PC2 axis also shows that Group 1 and the other three groups have significant differences in bacterial community structure (*p* < 0.05). The results of fungal community structure analysis are shown in Fig. 2B, where the two principal axes PC1 and PC2 show 19.38% and 12.00% of the overall variation, respectively. The scatter plot exhibits a clear separation trend in the distribution of the sample points between the HC group and the other three groups. Similarly, the box plot of PC2 axis also confirms the significant differences (*p* < 0.05) in fungal community structure between the HC group and the other three groups.

### Composition and structure of upper respiratory microbiota community

**Fig. 3:**
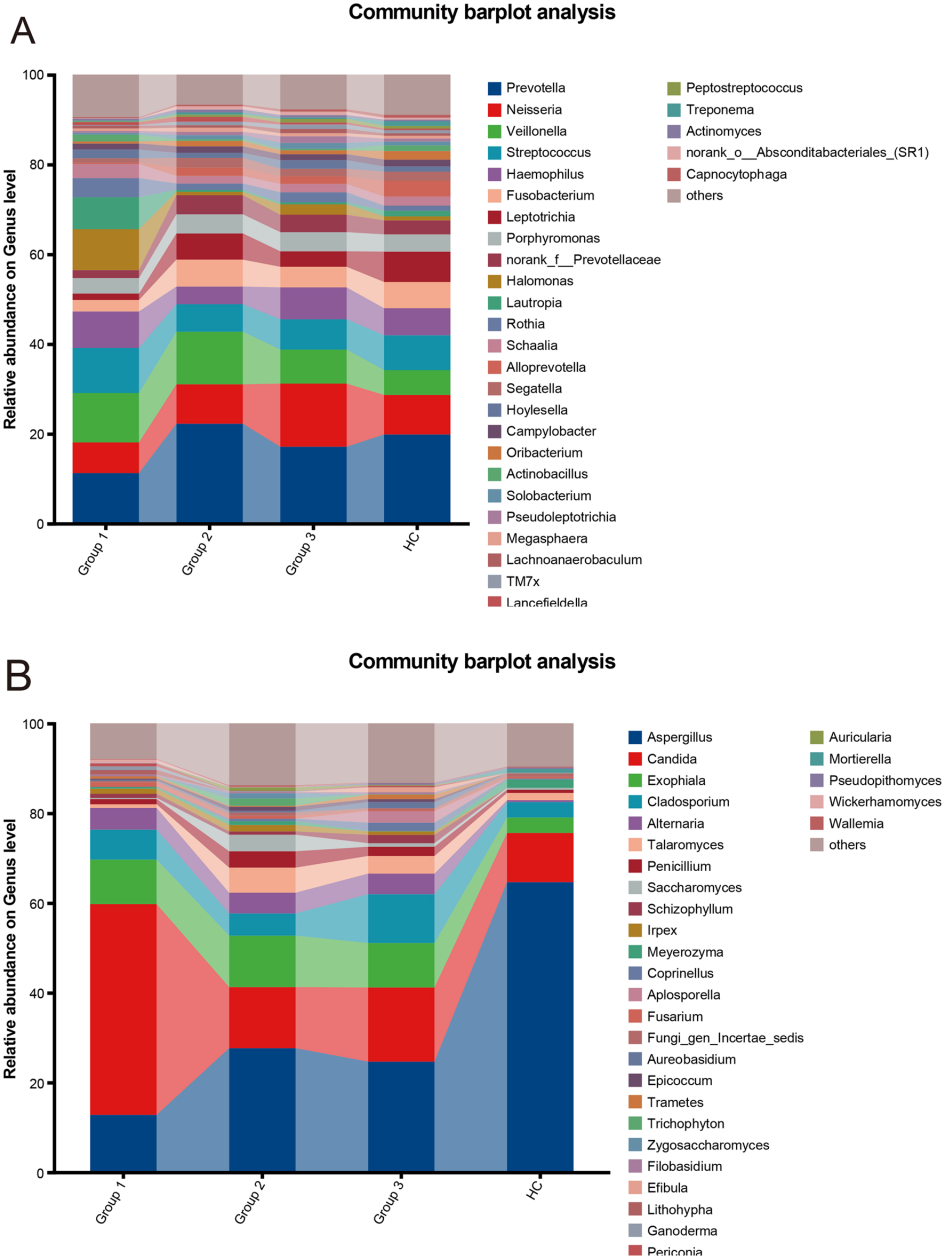
Relative abundance of upper respiratory microbiota in HIV infected individuals with different immune states and healthy controls. **A**: Bacterial community composition in the upper respiratory tract at the genus level. **B**: Fungal community composition in the upper respiratory tract at the genus level. For visualization purposes, the stacked bar plots display the 10 genera with the highest mean relative abundance in each group, and all remaining genera are grouped as “Others”. Different colors represent different bacterial or fungal genera. All detected taxa (at the ASV and genus levels) were included in the diversity and differential abundance analyses (see Methods)

Based on the relative abundance of bacteria and fungi at the genus level, we analyzed the overall structure of upper respiratory microbiota communities among different groups (Fig. 3). For the bacterial community (Fig. 3A), there were abundance differences among groups related to HIV immune status. Specifically, *Prevotella* and *Neisseria* have the lowest relative abundance in Group 1, which has the lowest immune function; The relative abundance of *Veillonella* and *Streptococcus* in the HIV infected groups (Group 1, 2, 3) was significantly higher than that in the healthy control (HC). In the fungal community (Fig. 3B), the relative abundance of *Aspergillus* in the HC group was significantly higher than in the three HIV infected groups. Meanwhile, *Candida* had the highest relative abundance in Group 1, which had the lowest immune function, significantly higher than the other three groups. In addition, the relative abundance of Exophiala in the HC group was lower than that in the HIV infected groups. Although Fig. 3 summarizes microbial composition at the genus level and displays only the 10 most abundant genera for clarity, all detected bacterial and fungal taxa (at the ASV and genus levels) were included in the statistical analyses, and the overall patterns of community shifts were consistent across taxonomic resolutions.

### Differential abundance analysis and core microbiome profiling of the upper respiratory tract

**Fig. 4:**
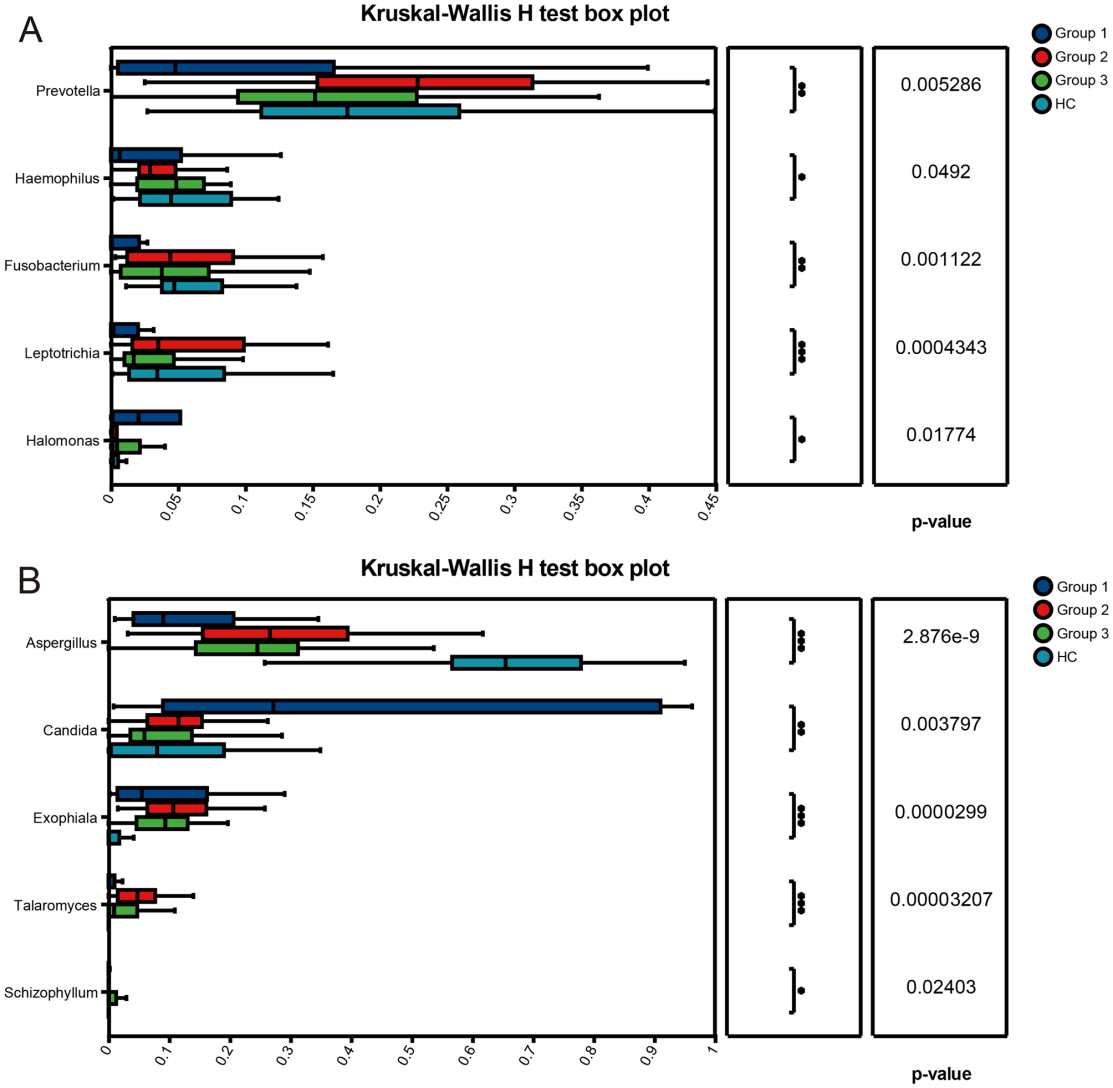
Comparison of core microbiome taxa in the upper respiratory tract between HIV infected individuals with different immune states and healthy controls. Core taxa were defined using both prevalence (occurrence across samples) and mean relative abundance thresholds. (**A**) Top 5 **core** bacterial genera ranked by mean relative abundance across groups. (**B**) Top 5 **core** fungal genera ranked by mean relative abundance across groups. Statistical differences in relative abundance across groups were assessed using Kruskal–Wallis tests with appropriate post-hoc comparisons, and p-values were adjusted for multiple testing using Benjamini–Hochberg FDR; only FDR-adjusted *p* < 0.05 was considered statistically significant. The significance level was defined as: *P* < 0.05, *P* < 0.01, *P* < 0.001; 0.01 < *P* ≤ 0.05 is marked as *, 0.001 < *P* ≤ 0.01 is marked as **, and *P* ≤ 0.001 is marked as ***

To identify taxa associated with immune status, we first conducted differential abundance testing based on relative abundance data across groups for all detected bacterial and fungal taxa, with Benjamini–Hochberg FDR correction as described in Methods. In addition, to provide a more conservative summary and reduce the number of taxa subjected to hypothesis testing, we performed a complementary core microbiome–based analysis. Following published core microbiome frameworks, the core microbiome was defined using both prevalence (occurrence across samples) and abundance, retaining taxa present in at least a prespecified proportion of samples (prevalence threshold) and exceeding a minimum mean relative abundance threshold. The total numbers of bacterial and fungal genera and species detected in each group, as well as the numbers retained after core filtering (based on prevalence ≥ 50% and mean relative abundance ≥ 0.1%), are summarized in Supplementary Table S1. Results of the core microbiome comparison are shown in Fig. 4. In the bacterial community (Fig. 4A), Group 1 (severe immunosuppression group) exhibits a unique distribution of bacterial genera. Compared with Group 2 (moderate immunosuppression group), Group 3 (no/mild immunosuppression group), and HC (healthy control group), the relative abundance of multiple common bacterial genera including *Prevotella*, *Haemophilus*, *Fusobacterium*, and *Leptotrichia* in Group 1 was significantly reduced. On the contrary, the relative abundance of *Halomonas* is significantly higher in Group 1 than in all the other groups. The inter-group difference pattern of fungal communities appears more complex (Fig. 4B), with a significantly higher abundance of *Aspergillus* in the HC group compared to all HIV infected groups (Groups 1, 2, 3). The abundance of *Exophiala* was significantly higher in all HIV infected groups than in the HC group. In addition, although *Talaromyces* and *Schizophyllum* have lower overall abundance, they show higher relative abundance in Group 2 and Group 3, respectively.

## Discussion

While the gut microbiome in HIV/AIDS has been extensively studied [4–21], characterization of the upper respiratory tract microbiota in relation to host immune status remains relatively limited. Elucidating the dynamics of the upper respiratory microbiota is of considerable importance, given its potential role in disease progression among people living with HIV. This study provides a systematic analysis of the upper respiratory microbiome and demonstrates that host immunosuppression level is strongly associated with microbial diversity, community structure, and the abundance of specific bacterial and fungal taxa—even after adjusting for behavioral and environmental confounders such as smoking, oral hygiene practices, recent antimicrobial exposure, dietary habits, and periodontal disease.

Our results show that bacterial α-diversity in the upper respiratory tract is inversely correlated with the severity of immune suppression. Specifically, individuals in the severe immunosuppression group (CD4⁺ T-cell count < 200 cells/µL) exhibited significantly lower bacterial richness and diversity compared with all other groups. This finding aligns with earlier reports of reduced microbial diversity in the gut and respiratory tract of HIV-infected individuals [22–24]. Declining bacterial diversity is widely regarded as a hallmark of dysbiosis [9], and likely reflects compromised local immune surveillance, permitting overgrowth of certain taxa and destabilization of the indigenous symbiotic ecosystem [23].

In contrast, fungal community richness remained relatively consistent across all groups, suggesting that overall fungal load is not markedly influenced by HIV infection or immune status. Notably, although fungal α-diversity was significantly lower in the severe immunosuppression group compared to other HIV-positive groups, it did not differ statistically from healthy controls—a result consistent with the observations of Fukui et al. [14]. This suggests that reductions in fungal diversity may not be directly attributable to HIV-associated immunosuppression, but could be related to other factors operative in advanced disease. Interestingly, fungal diversity was significantly elevated in individuals with moderate or mild immunosuppression. This may reflect an initial phase of immune decline in which altered host conditions permit broader fungal colonization. As disease advances and immunosuppression becomes severe, however, this transiently expanded fungal community may collapse, leading to pronounced diversity loss.

PCoA of beta diversity revealed distinct shifts in community structure, with bacterial assemblages displaying patterns of association in contrast to those observed for fungi. For bacteria, the main separation occurred between the severe immunosuppression group and all other groups, mirroring the α-diversity results. We hypothesize that this bacterial dysbiosis is strongly linked to progressive immune deterioration as individuals transition to AIDS. In contrast, fungal community structure did not segregate clearly by immune status; instead, the most pronounced differentiation was observed between healthy controls and all HIV-infected groups. This implies that HIV infection itself may trigger a durable shift in the upper respiratory fungal community—a conclusion supported by compositional analyses showing consistent differences in *Aspergillus* and *Exophiala* abundance between HIV-positive individuals and controls. The lack of significant change in fungal richness further suggests that HIV-induced alterations involve redistribution of relative abundances rather than loss of taxa.

Analysis of core bacterial genera in the upper respiratory tract showed that several commensals—including *Prevotella*, *Fusobacterium*, *Leptotrichia*, and *Haemophilus*—were significantly depleted in the severe immunodeficiency group, consistent with a previous report from Japan [14]. HIV infection is known to cause mucosal immune injury, and the associated pro-inflammatory milieu may further suppress the growth of these symbiotic bacteria [4]. Moreover, multiple studies on upper respiratory microbiota have documented decreased abundance of *Fusobacterium* and *Leptotrichia* in the context of HIV or other viral infections [25, 26]. Conversely, *Halomonas* was markedly enriched in the severe immunodeficiency group. This genus is commonly isolated from marine and industrial nitrification environments, raising the possibility that local environmental exposure or geographic factors contributed to its prominence in our cohort [27]. However, as environmental samples were not collected and comparative multi-site data are lacking, the potential influence of geography remains speculative and warrants verification in future studies.

Regarding the fungal component, our analysis revealed that *Aspergillus* was significantly more abundant in healthy controls than in any of the HIV-infected groups. This finding contrasts with much of the existing literature, which generally reports either no significant difference in *Aspergillus* abundance between HIV-infected individuals and healthy controls, or even elevated levels in the infected population [28]. The discrepancy observed here may reflect regional variations or methodological differences related to sampling sites.

More notably, this study identified colonization patterns of two clinically relevant opportunistic pathogenic fungi. First, *Exophiala*—a genus containing several species known to cause invasive respiratory infections [29]—was consistently enriched across all HIV-infected groups compared to healthy controls. This widespread colonization suggests that even individuals with preserved immune function may serve as reservoirs for such fungi, posing a potential risk for invasive disease should immune competence further decline.

The most clinically salient finding was the specific enrichment of Candida in the severe immunodeficiency group, a pattern consistent with clinical evidence identifying oropharyngeal candidiasis as one of the most frequent opportunistic infections in AIDS [30–32]. Given that this study assessed fungal colonization rather than active clinical infection, these findings suggest that profound immunosuppression fosters a permissive environment for Candida overgrowth, thereby increasing susceptibility to oropharyngeal candidiasis and related opportunistic conditions. Accordingly, elevated pharyngeal Candida abundance may be more accurately viewed as a manifestation of local dysbiosis linked to severe immune impairment, rather than as a diagnostic biomarker for established disease.

Several limitations of this study should be acknowledged. First, the relatively small size of the healthy control group may have limited the statistical power of β-diversity and differential abundance analyses; therefore, these results should be interpreted with caution. Second, although all HIV-infected participants were receiving ART, detailed data on treatment duration, specific regimens, adherence, or virologic failure history were not collected. These factors are known to affect microbiome composition and could represent important confounders. Third, while recent systemic antimicrobial exposure was recorded and adjusted for in multivariable models, detailed information on specific agents, dosages, and treatment durations was unavailable, leaving the possibility of residual confounding.

In summary, our findings demonstrate that bacterial and fungal communities in the upper respiratory tract follow distinct trajectories following HIV infection. Bacterial community disturbance correlates strongly with the degree of host immunosuppression, with marked reductions in α-diversity and shifts in β-diversity most evident in the severe immunodeficiency group—highlighting a strong association between bacterial ecosystem stability and host immune function. In contrast, fungal community structure appears to be influenced more by HIV infection status itself.

## Supplementary Information

Below is the link to the electronic supplementary material.

Supplementary Material 1

Supplementary Material 2

## Data Availability

Raw sequencing data have been uploaded to the NCBI Sequence Read Archive (SRA) under the BioProject accession number PRJNA1332397.
