# Supplementary material for: Analysis of pharyngeal microbiome characteristics in HIV-infected individuals: correlation between the degree of immunosuppression and microbial dysbiosis
Source: BMC Infect Dis. 2026 Mar 24;26:877. doi: 10.1186/s12879-026-13075-2 (PMC13137539; doi:10.1186/s12879-026-13075-2)
Supplement: Supplementary file 1 — Supplementary Material 1 [file 12879_2026_13075_MOESM1_ESM.docx]

**Research Participant Information Registration Form**​
**Study ID:​**​ ____________________ ​ **Registration Date:​**​ _____________________

**Group:​**​ □ PLWH Group □ Healthy Control Group

​

**Part 1: Eligibility Confirmation**​

1. Have you read the informed consent form and voluntarily agreed to participate in this study?
   □ Yes
   □ No
2. Are you 18 years of age or older?
   □ Yes
   □ No
3. Are you currently experiencing acute respiratory symptoms?
   □ Yes
   □ No

​**Part 2: Basic Information**​

1. Year of Birth: 19____
2. Gender:
   □ Male
   □ Female

​**Part 3: Clinical Information for PLWH**​
*(Healthy controls do not need to complete this section)*

1. Have you started antiretroviral therapy?
   □ Yes
   □ No
2. Have you undergone CD4+ T-cell count and viral load testing in the past three months?
   □ Yes
   □ No
3. Your most recent CD4+ T-cell count is approximately: _______________ cells/μL
4. Your most recent plasma viral load is approximately: ________________ copies/mL

​

**Investigator’s Signature:​**​ _________________________ ​ **Date:​**​ _________________________
