# Supplementary material for: Analysis of pharyngeal microbiome characteristics in HIV-infected individuals: correlation between the degree of immunosuppression and microbial dysbiosis
Source: BMC Infect Dis. 2026 Mar 24;26:877. doi: 10.1186/s12879-026-13075-2 (PMC13137539; doi:10.1186/s12879-026-13075-2)
Supplement: Supplementary file 2 — Supplementary Material 2 [file 12879_2026_13075_MOESM2_ESM.doc]

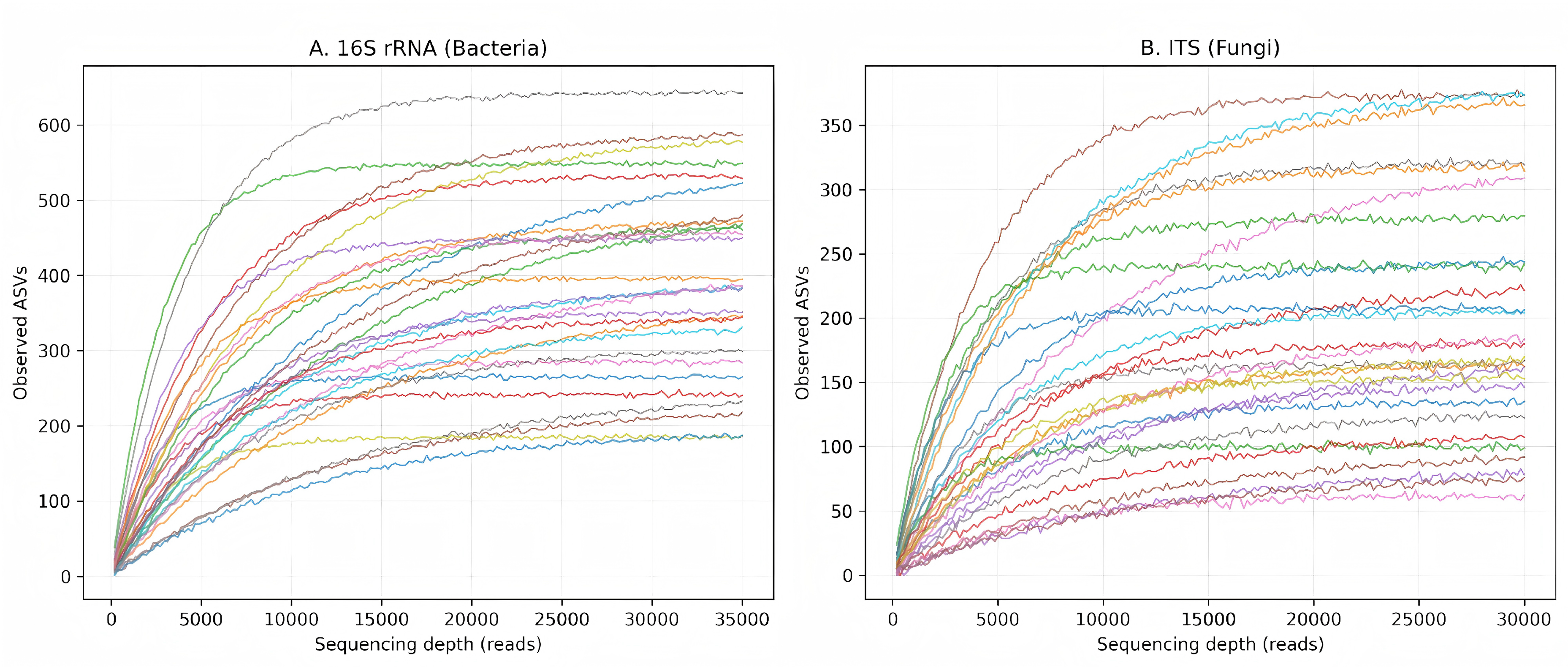


Supplementary Figure S1. Rarefaction curves of bacterial (16S rRNA) and/or fungal (ITS) communities. Curves show the relationship between sequencing depth and observed ASV richness for each sample, indicating sampling adequacy when curves approach saturation.

Supplementary Table S1. Taxonomic summary and core microbiome filtering results

| **Microbial Domain** | **Taxonomic Level** | **Group** | **Total Detected Taxa** | **Core Threshold (Prevalence ≥50%, Mean RA ≥0.1%)** | **Core Taxa Retained** |
| --- | --- | --- | --- | --- | --- |
| Bacteria | Genus | Group 1 (CD4<200) | 123 | Yes | 34 |
| Bacteria | Genus | Group 2 (200<CD4<500) | 119 | Yes | 32 |
| Bacteria | Genus | Group 3 (CD4>500) | 125 | Yes | 35 |
| Bacteria | Genus | Healthy Controls (HC) | 111 | Yes | 30 |
| Bacteria | Species | Group 1 | 86 | Yes | 21 |
| Bacteria | Species | Group 2 | 80 | Yes | 19 |
| Bacteria | Species | Group 3 | 89 | Yes | 22 |
| Bacteria | Species | HC | 74 | Yes | 17 |
| Fungi | Genus | Group 1 | 57 | Yes | 12 |
| Fungi | Genus | Group 2 | 54 | Yes | 11 |
| Fungi | Genus | Group 3 | 59 | Yes | 13 |
| Fungi | Genus | HC | 52 | Yes | 10 |
| Fungi | Species | Group 1 | 38 | Yes | 8 |
| Fungi | Species | Group 2 | 35 | Yes | 7 |
| Fungi | Species | Group 3 | 41 | Yes | 9 |
| Fungi | Species | HC | 33 | Yes | 6 |
